# Revealing Probiotic Potential of *Enterococcus* Strains Isolated From Traditionally Fermented *Chhurpi* and Healthy Human Gut

**DOI:** 10.3389/fmicb.2022.909987

**Published:** 2022-06-16

**Authors:** Kriti Ghatani, Subarna Thapa, Shankar Prasad Sha, Sourav Sarkar, Debabrata Modak, Soumen Bhattacharjee

**Affiliations:** ^1^Food Microbiology Laboratory, Department of Food Technology, University of North Bengal, Raja Rammohunpur, India; ^2^Food Microbiology Laboratory, Department of Botany, Kurseong College, Kurseong, India; ^3^Cell and Molecular Biology Laboratory, Department of Zoology, University of North Bengal, Raja Rammohunpur, India

**Keywords:** *chhurpi*, human gut, probiotic, *Enterococcus*, blood lipids, histopathology

## Abstract

In this study, the two lactic acid bacterial strains *Enterococcus durans* and *Enterococcus lactis* previously isolated from soft *chhurpi*, a traditionally fermented milk product prepared by the indigenous community of Sikkim Himalayas and healthy human gut were used. In this study, we attempted to investigate the probiotic attributes, safety, and health beneficial role, and hypercholesterolemia of *Enterococcus durans* and *Enterococcus lactis*. Both probiotic potential strains showed good hypocholesterolemic activity *in vitro* along with tolerance to acid pH (2 and 2.5), tolerance to three bile salts, oxbile, cholic acid, and taurocholic acid (0.5 and 1%), presence of BSH enzyme and its activity, and cell surface adherence. On assessing for safety, both LAB strains were sensitive to antibiotics and exhibited no hemolytic activity. The probiotic strains were tested *in vivo* in the Sprague–Dawley rats which were divided into five experimental groups: Normal Control (ND), probiotic strain *Enterococcus durans* HS03 (BSH-negative) and high-cholesterol diet (HCD1), probiotic strain *Enterococcus lactis* YY1 (BSH-positive) and high-cholesterol diet (HCD2), and a combination of both strains and high-cholesterol diet (HCD3) and Negative Control (HCD). The probiotic-treated groups HCD1, HCD2, and HCD3 showed a decrease in serum cholesterol levels up to 22.55, 6.67, and 31.06%; the TG and VLDL concentrations were 25.39, 26.3, and 33.21%; reduction in LDL-cholesterol was 33.66, 28.50, and 35.87%; and increase of HDL was 38.32, 47.9, and 41.92%. Similarly, the effects of total cholesterol and TG in the liver, kidney and liver histopathology, liver and body lipid index, and oxidative stress in rat liver were also studied. The fecal lactobacilli were more in the samples of the probiotic-treated groups and their fecal coliform and *E*. *coli* counts decreased relatively as compared to the control groups in 0, 7, 14, and 21 days. This is the first report on the probiotic potential of *Enterococcus durans* HS03 and *Enterococcus lactis* YY1 strains that gives a new insight into the cholesterol-lowering and probiotic product development with wide health attributes.

## Introduction

Over the last decades, the ubiquity of hyperlipidemia among people throughout the world has inflated and attained an epidemic level (Zhang et al., 2021). Hyperlipidemia is a condition in which the plasma or serum contains elevated levels of total cholesterol (TC) and/or triglycerides (TG) and/or low-density lipoprotein (LDL); and low levels of high-density lipoprotein (HDL) (Wang et al., 2021). Numerous aspects like environmental factors, unhealthy food habits, and heredity may be the cause of hyperlipidemia. Whatever be the causative factors of hyperlipidemia, long-term exposure to such a condition as hyperlipidemia may be followed by various health issues like cardiovascular diseases, cerebrovascular diseases, atherosclerosis, metabolic syndrome, and many chronic diseases (Skrypnik et al., 2018; Wang et al., 2021). Although the use of drug therapy (statins, fibrate, etc.) has proved promising for the treatment of hyperlipidemia. These drugs are associated with various side effects such as diarrhea, abdominal discomfort, flatulence, dementia, low levels of vitamin D in the plasma, and sleep deprivation and are also very expensive (Wang et al., 2012; Ma et al., 2017; Zhang et al., 2021). Further, the continuous use of statins may lead to liver damage and muscle toxicity (Zhang et al., 2021). Considering such effects of hyperlipidemia and also the side effects of drug therapy, today, much focus has been given to the ability of probiotics as an alternative strategy to curb the incidence of hyperlipidemia *via* various mechanisms (Tomaro-Duchesneau et al., 2014). The mechanisms involved in the hypolipidemic activity of probiotics have been suggested to involve the deconjugation of bile acid *via* bile salt hydrolase activity, cholesterol binding to the probiotic cell wall, transformation of the cholesterol into coprostanol, redistribution of the plasma cholesterol to the liver, inhibition of hepatic cholesterol and triglyceride synthesis by short-chain fatty acids (e.g., propionate), and assimilation of cholesterol (Salaj et al., 2013).

Probiotics are “live microorganisms”, mostly belonging to the lactic acid bacterial group, and include species of *Lactobacillus, Bifidobacterium, and Enterococcus*, that have become propitious agents to confer health benefits to the host (Hotel and Cordoba, 2001; Zhu et al., 2019). Probiotics have proven as promising agents for decreasing the blood LDL and TC concentrations (Skrypnik et al., 2018; Nami et al., 2019). The earliest documented case of serum cholesterol reduction by probiotic *Lactobacillus* strain was reported by Shaper et al. (1963); Mann and Spoerry (1974) and Vinderola and Reinheimer (2003). An *in vivo* study conducted by Guo et al. (2016) on the effect of oral feeding of *Enterococcus durans* in hypercholesterolemic Sprague–Dawley rats suggested the potential of *E*. *durans* to lower total serum cholesterol and LDL (Guo et al., 2016). *In vitro* study by Albano et al. (2018) on the hypocholesterolemic effect of *Enterococcus lactis* BT161 has reported a 42–55% decrease in the cholesterol level in the broth (Albano et al., 2018).

Although numerous strains of *Bifidobacterium* and *Lactobacillus* species have been reported to alleviate cholesterol both *in vivo* and *in vitro*, very limited reports are available for the hypocholesterolemic effect of the *Enterococcus* strains (Tomaro-Duchesneau et al., 2014; Shehata et al., 2016; Hyrslova et al., 2021; Marras et al., 2021; Yang et al., 2021; Kouhi et al., 2022). Thus, this study has aimed to determine the beneficial effects of probiotic *Enterococcus durans* HS03 (BSH-negative) and *Enterococcus lactis* YY1 (BSH-positive) on lipid metabolism in Sprague–Dawley rats fed with a high-cholesterol diet.

## Materials and Methods

### Bacterial Strain and Culture Conditions

*Enterococcus durans* HS03 (accession no. KX274030) and *Enterococcus lactis* YY1 (accession no. KU 601443) were the two strains used in the study. These isolates were previously isolated from the human gut and *chhurpi* (fermented yak milk product) and examined for cholesterol-lowering property in the laboratory (Ghatani and Tamang, 2017). The *chhurpi* samples were collected from North Sikkim. Yak (*Bos gruinnens*), known as *gyag* in the Bhutia language, is a multipurpose “sure-footed animal” of the Himalayan region. Several value-added products are made from yak milk, meat, skin, hair, and horns (Ghatani and Tamang, 2016). Yak milk and its products are popular foods in high-altitude regions and it plays a major role in providing essential nutrients and minerals to the herdsman and the local people. The most common products made from milk are *marr* (Ghew in Nepali), *chhurpi, thara* (Mohi in Nepali), *shyow* (Dahi in Nepali), *chilu* (yak fat), *philu* (Dewan and Tamang, 2007), and *tema* (yak cream) (Ghatani and Tamang, 2016). There are three different types of *chhurpi*: hard type, soft type, and *dudh chhurpi*. The strain *Enterococcus lactis* YY1 has been isolated from the soft variety that is prepared by boiling buttermilk or whey to form a white mass that is sieved out from the remaining liquid (Ghatani and Tamang, 2017).

The strains were subcultured in de Man, Rogosa, and Sharpe (MRS) broth (Hi Media, Mumbai, India) followed by incubation at 37°C in an Anaerobic Gas Pack system (Hi Media LE002, Mumbai, India) for 72 h.

### Tolerance to Low pH/Acid

The overnight-grown culture 1% was inoculated in MRS broth supplemented with 0.30% oxgall, pH adjusted to 2.5 with HCl, and incubated at 37°C for 2 h (Liong and Shah, 2005). Serial dilutions were prepared and plated every 30 min until 2 h in MRS agar. The strains were tested for acid tolerance at pH 2 determined by plate count methods.

### Tolerance to Bile Salt

The strains were tested for tolerance to three different types of bile salts—oxgall, cholic acid, and taurocholic acid (Liong and Shah, 2005) with slight modifications. MRS broths containing 0.5 and 1% (w/v) of oxgall, cholic acid, or taurocholic acid were inoculated with each strain and incubated at 37°C. MRS broth without bile salt was used as a control according to the method of Gilliland and Walker (1990). Bacterial growth was monitored by measuring the turbidity with a spectrophotometer (Lambda UV-VIS spectrophotometer, Perkin Elmer, Wokingham, United Kingdom) at 620 nm at 0 and 8 h, respectively.

### Cell Surface Hydrophobicity

The strains were grown in MRS broth at 37°C for 24 h and centrifuged at 8,000 g for 5 min (Rosenberg, 1984; Pérez et al., 1998), the pellet was washed three times with Ringer solution (Merck, Darmstadt, Germany), and thoroughly mixed in a vortex; 1 ml of the suspension was taken and the absorbance at 580 nm was measured. Then 1.5 ml of the suspension was mixed with an equal volume of n-hexadecane (RM 2238, HiMedia, Mumbai, India) in duplicates and mixed thoroughly in a vortex. The phases were allowed to separate for 30 min at room temperature, after which the aqueous phase was carefully removed and absorbance at 580 nm was measured (Nami et al., 2020; Kiani et al., 2021).

### Direct Plate Assay for Screening BSH Activity

The strains were checked for bile salt hydrolase activity by the direct plate assay technique with slight modifications by Nguyen et al. (2007). The cultures were streaked on MRS agar plates containing 0.5% (w/v) taurodeoxycholic acid sodium salt (TDCA: Sigma) and incubated in an anaerobic jar at 37°C for 72 h. Only MRS agar plates were streaked for comparison. After incubation, the presence of precipitated bile acid surrounding colonies (opaque halo) or the formation of opaque granular white colonies with a silvery-white shine was considered as the presence of BSH enzyme.

### BSH Assay

BSH activity was measured by determining the number of amino acids liberated from conjugated bile salts by lactobacilli strains as described by Tanaka et al. (1999), with few modifications.

### Safety Evaluation of Probiotics for Human Use

Probiotic bacteria must be studied for the following parameters to be generally recognized as safe. So, the strains were tested for safety before *in vivo* study.

Determination of antibiotic resistance patterns for any risk of transferable antibiotic resistance. Antibiotic resistance patterns were studied using antibiotic disk of penicillin (*P*) 10 units, erythromycin (E) 15 μg, vancomycin (VA) 30 μg, teicoplanin (TE1) 30 μg, clindamycin (CD) 2 μg, ofloxacin (OF) 5 μg, azithromycin (AZM) 15 μg, and tetracycline (TE) 30 μg (Kumar et al., 2017). The *E coli* MTCC 1098 and *Staphylococcus aureus* MTCC 7443 were used as control strains for antibiotic susceptibility.

Hemolytic activity was investigated as described by Gerhardt et al. (1913) by incubating blood agar plates (48 h at 37°C) and examining them for the signs of β-hemolysis (clear zones around colonies), α-hemolysis (green zones around colonies), or γ hemolysis (no clear zones around colonies).

### *In vivo* Study

#### Experimental Animals

A total of 30 adult male Sprague–Dawley (SD) rats (200 ± 10 gm) were purchased from CSIR-CDRI (Central Drug Research Institute), Lucknow, India. All animals were housed in well-ventilated polypropylene cages (Tarsons, India) with paddy husk as bedding material in the Animal House of Department of Zoology, University of North Bengal. All the animals were maintained under a constant 12 h light−12 h dark cycle. Room temperature was controlled at 22–25°C with about 56–60% relative humidity. The animals were provided with sufficient food and water *ad libitum*. The animals undergo acclimatization for 1 week to the laboratory environment before the start of the experiment. The approval of the Institute's Animal Ethics Committee (IAEC/NBU/2019/17) was obtained before conducting the experimental trial from CPCSEA (Committee for the Purpose of Control and Supervision of Experiments on Animals) of the University of North Bengal, West Bengal, India.

#### Animal Diet

AIN-76 mineral mixture (MP Biomedicals, France) with 1% cholesterol was used as the high-cholesterolemic diet. The base composition of the experimental AIN-76 diet is recorded in Table 1. However, the normal diet used in the experiment does not include cholesterol in its high-cholesterolemic diet (Kumar et al., 2011).

**Table 1:** Composition of experimental high- cholesterolemic diet.

**Constituents**			**In %**
High-cholesterolemic diet	AIN-76 semi purified diet	Corn starch	15
		Casein purified high Nitrogen	20
		Corn oil	5
		Vitamin mixture	1
		Mineral mixture	3.5
		DL-methionine	0.30
		Cholesterol	0.50
		Sucrose	50
		Choline bitartrate	0.2
		Alphacel, non-nutritive bulk	5
	Cholesterol	Cholesterol	1

#### Animal Grouping

All the 30 rats were divided into 5 experimental groups having 6 rats each (*n* = 6). The experimental groups included are summarized in Table 2. The HCD group contained 1% (w/w) supplemental cholesterol.

**Table 2:** Experimental grouping used in this study.

**SL. No**.	**Group**	**Experimental diets**
1.	Normal control (ND)	Normal diet without cholesterol (AIN76)
2.	Probiotic strain 1 (HCD1)	High-cholesterol diet (AIN76+ 1% cholesterol) + *Enterococcus durans* HS03 (~10^8^ cfu/g)
3.	Probiotic strain 2 (HCD2)	High-cholesterol diet (AIN76+ 1% cholesterol) + *Enterococcus lactis*YY1 (~10^8^ cfu/g)
4.	Probiotic strain 1 and 2 (HCD3)	High-cholesterol diet (AIN76+ 1% cholesterol) + *Enterococcus durans* HS03 and *Enterococcus lactis*YY1 (~10^8^ cfu/g)
5.	Negative control (HCD)	High-cholesterol diet (AIN76+ 1% cholesterol)

### Preparation of Bacterial Cultures

The probiotic strains were all grown in an MRS medium under anaerobic conditions. The overnight-grown test cultures were pelleted at 5,000 rpm for 30 min at 4°C and washed two times with saline. The strains were resuspended at 1 × 10^8^ CFU/ml concentration in neutral saline. The number of CFU administered was routinely verified by plating. The culture was concentrated to 20 ml (Stock) and kept at 8°C until feeding; 0.3 ml bacterial solution was administered to each rat by intragastric gavage (Kumar et al., 2011).

### Feeding Schedule

The rats were fed with standard pellets for 7 days to remove the effect of stress during the acclimatization phase. At the end of the acclimatization phase, all the groups were fed their respective experimental diets for the next 21 days. During the entire course of the experiment, the rats had free access to water. Each rat was fed with 10 gm/day of diet from the 1st week, which was then increased by 2 gm/day per week. Feed intake was recorded daily and body weight was measured weekly.

### Blood Sampling and Serum Lipid Profile Analysis

After the treatment schedule, all animals were anesthetized with sodium pentobarbital (60 mg/kg; i.p.) and euthanized by cervical dislocation (Modak et al., 2021). Thereafter, blood samples were obtained through the cardiac puncture placed in sterile microfuge tubes. For serum analyses, blood samples were centrifuged at 5,000 rpm for 10 min at 4°C and serums were collected. The samples were analyzed for total cholesterol (TC), triglycerides (TG), LDL-cholesterol (LDL-C), and HDL-cholesterol (HDL-C) using commercial Coral Kits (Coral clinical systems, India) following the manufacturers' protocol with a spectrophotometer (Lambda UV-VIS spectrophotometer, Perkin Elmer, Wokingham, United Kingdom). The Atherogenic index (AI) and VLDL-cholesterol were calculated as per Friedewald's equation (Friedewald et al., 1972).

### Assay for Liver Lipids

Liver tissues (100 mg) were pulverized in liquid nitrogen to prepare 10% tissue homogenate. These homogenates were extracted under 4°C with 5 ml of Folch solution (chloroform: methanol ratio = 2:1) for 48 h, then centrifuged at 10,000 rpm for 15 min at 4°C. The concentrations of liver TG and TC in the supernatant were determined according to the protocols (Yin et al., 2010).

### Liver and Kidney Histopathology

After euthanization, the liver and kidney tissues of each rat from each group were immediately removed and washed in chilled phosphate buffer saline (pH 7.4). The middle lobe of the liver was standardized as the sampling region. Both the tissues were sectioned and soaked in 4% formalin for fixation and were dehydrated using serial dilutions of ethanol and subsequently embedded in paraffin wax according to previous protocols (Modak et al., 2021). The tissues were cut into transverse sections at 5 μm thickness and stained with hematoxylin and eosin. Thereafter, microscopic examinations were done under a light microscope (Nikon Eclipse E200, Nikon, Tokyo, Japan) with 10X and 40X objectives and, respectively, scale bar had been attached (100 and 25 μm). Each histologic section was observed for 5 fields of high-power fields.

### Assay for Liver and Body Lipid Index

After euthanization, the liver and white adipose tissue (WAT) of the mesenteric fat-pad and epididymal fat-pad of each rat from each group were immediately removed and weighed. Measurements were (1) liver index: liver weight/body weight ratio and (2) body lipid index: viscera fat weight/body weight ratio [viscera fat includes retroperitoneal (RET) and epididymal (EPI) white adipose tissues] (Yin et al., 2010).

### Preparation of Liver Homogenate and Analysis of Oxidative Stress Markers

The liver homogenate was prepared following standard protocol. Briefly, liver tissue was removed from each rat at the time of sacrifice and 10% of liver homogenates was obtained by homogenizing liver tissues (1 gm) in 10 ml cold phosphate buffer (pH 7.4) solution (4°C). The homogenates were centrifuged at 12,000 rpm for 10 min at 4°C and the resulting supernatant was collected and used to detect oxidative stress markers.

### Determination of Lipid Peroxidation

Lipid peroxidation in the liver was estimated colorimetrically by measuring the thiobarbituric acid reactive substrates (TBARS) using a previously described method (Draper and Hadley, 1990). The absorbance of the clear supernatant was measured at 535 nm against a blank reference.

### Determination of Catalase

CAT activities were determined using a previously described method (Aebi, 1974). Changes in absorbance of the reaction solution at 240 nm were determined after 1 min. One unit of CAT activity was defined as a change in the absorbance of 0.01 unit/min.

### Determination of Reduced Glutathione

Reduced glutathione levels were estimated using the method reported by Ellman (1959). After the yellow color developed, the absorbance of the mixture was immediately recorded at 412 nm against a blank reference (Ellman, 1959).

### Microbiological Analysis of Fecal Samples

Fecal samples from distinctly grouped, experimental SD rats were collected in distinct sterile vials to analyze their microbial content. Each sample was homogenized in sterile PBS using a stomacher blender. This was followed by a ten-fold serial dilution of each sample in peptone water; 1 ml of each diluent was placed on nutrient agar, eosin methylene blue agar, violet red bile agar, and MRS agar. Nutrient agar, eosin methylene blue agar, violet red bile agar, and MRS agar were used for total plate count, *Escherichia coli*, coliforms, and total lactobacilli count, respectively. All the plates except those for the enumeration of total lactobacilli were incubated at 37°C for 48 h. The MRS agar plates for the enumeration of total lactobacilli were incubated under anaerobic conditions using a desiccator at 37°C for 48 h (Kumar et al., 2011).

### Statistical Analysis

For *in vitro* experiments, all parameters were repeated six times and were presented as mean ± SD. For *in vivo*, all data were presented as the mean ± SD (*n* = 6). However, the body and organ weight was measured as ±SEM. Univariate analysis of variance test was applied to determine the statistical significance of the difference among the groups, using the using Graph Pad Prism Version 7.00 (San Diego, United States of America). Comparison between more than two groups was done using one-way analysis of variance (ANOVA) or by two-way ANOVA following the *post-hoc* analysis with a Dunnett's multiple comparisons test. Values of *p* ≤ 0.05 were taken to indicate a statistical difference.

## Results

Lowering of cholesterol was observed in both the strains up to 70 and 65% for *Enterococcus durans* HS03 and *Enterococcus lactis* YY1, respectively (Ghatani and Tamang, 2017). The two strains were then tested for probiotics attributes like tolerance to acid, tolerance to bile, hydrophobic nature, and antibiotic sensitivity tests.

On checking *Enterococcus durans* HS03 and *Enterococcus lactis* YY1 for the ability to tolerate acidic conditions, first at pH 2.5 and then at pH 2.0, was found to be satisfactory. The pH of gastric juice secreted in the stomach is about 2 and many microorganisms are destroyed at this and lower pH. So, tolerance to acidic conditions is a very important criterion for the selection of probiotic bacteria. The pH 2.0 was regarded as a strong discriminative pH for the selection of high acid-tolerant strains. The viable cell counts (log CFU/ml) and survival percentages of selected LAB to acid conditions at pH 2 after 2 h incubation is presented in Table 3.

**Table 3:** Probiotic attributes of *Enterococcus durans* HS03 and *Enterococcus lactis* YY1.

**Sl. no**.	**Probiotic characters**	**Conditions**	**Duration**	**Strains**	
				***Enterococcus durans*** **HS03**	***Enterococcus lactis*** **YY1**
1.	Acid tolerance (viable count log CFU/ml)	pH 2.5	0 h	9.75 ± 0.30	10.13 ± 0.23
			1 h	5.11 ± 0.35	7.60 ± 0.23
			2 h	2.65 ± 0.21	5.17 ± 0.12
		pH 2	0 h	9.60 ± 0.34	9.67 ± 0.007
			1 h	5.01 ± 0.12	3.2 ± 0.01
			2 h	2.05 ± 0.31	1.5 ± 0.24
2.	Tolerance to bile (turbidity or tolerance)	Ox bile (0.5%)	0 h	0.13 ± 0.23	0.16 ± 0.01
			4 h	0.33 ± 0.34	0.19 ± 0.01
			8 h	0.49 ± 0.45	0.31 ± 0.11
		Taurocholic acid (0.5%)	0 h	0.14 ± 0.12	0.12 ± 0.25
			4 h	0.47 ± 0.11	0.34 ± 0.13
			8 h	1.56 ± 0.23	1.15 ± 0.02
		Cholic acid (0.5%)	0 h	0.13 ± 0.11	0.14 ± 0.12
			4 h	0.15 ± 0.23	0.17 ± 0.26
			8 h	0.21 ± 0.25	0.29 ± 0.34
		Ox bile (1%)	0 h	0.13 ± 0.34	0.17 ± 0.02
			4 h	0.23 ± 0.25	0.25 ± 0.19
			8 h	0.35 ± 0.23	0.33 ± 0.30
		Taurocholic acid (1%)	0 h	0.11 ± 0.23	0.13 ± 0.59
			4 h	0.33 ± 0.12	0.36 ± 0.56
			8 h	0.80 ± 0.11	1.40 ± 0.03
		Cholic acid (1%)	0 h	0.12 ± 0.54	0.12 ± 0.04
			4 h	0.16 ± 0.34	0.14 ± 0.06
			8 h	0.24 ± 0.14	0.22 ± 0.08
3.	Cell surface hydrophobicity (%)			65 ± 0.34	70 ± 0.25

**Figure 1:**
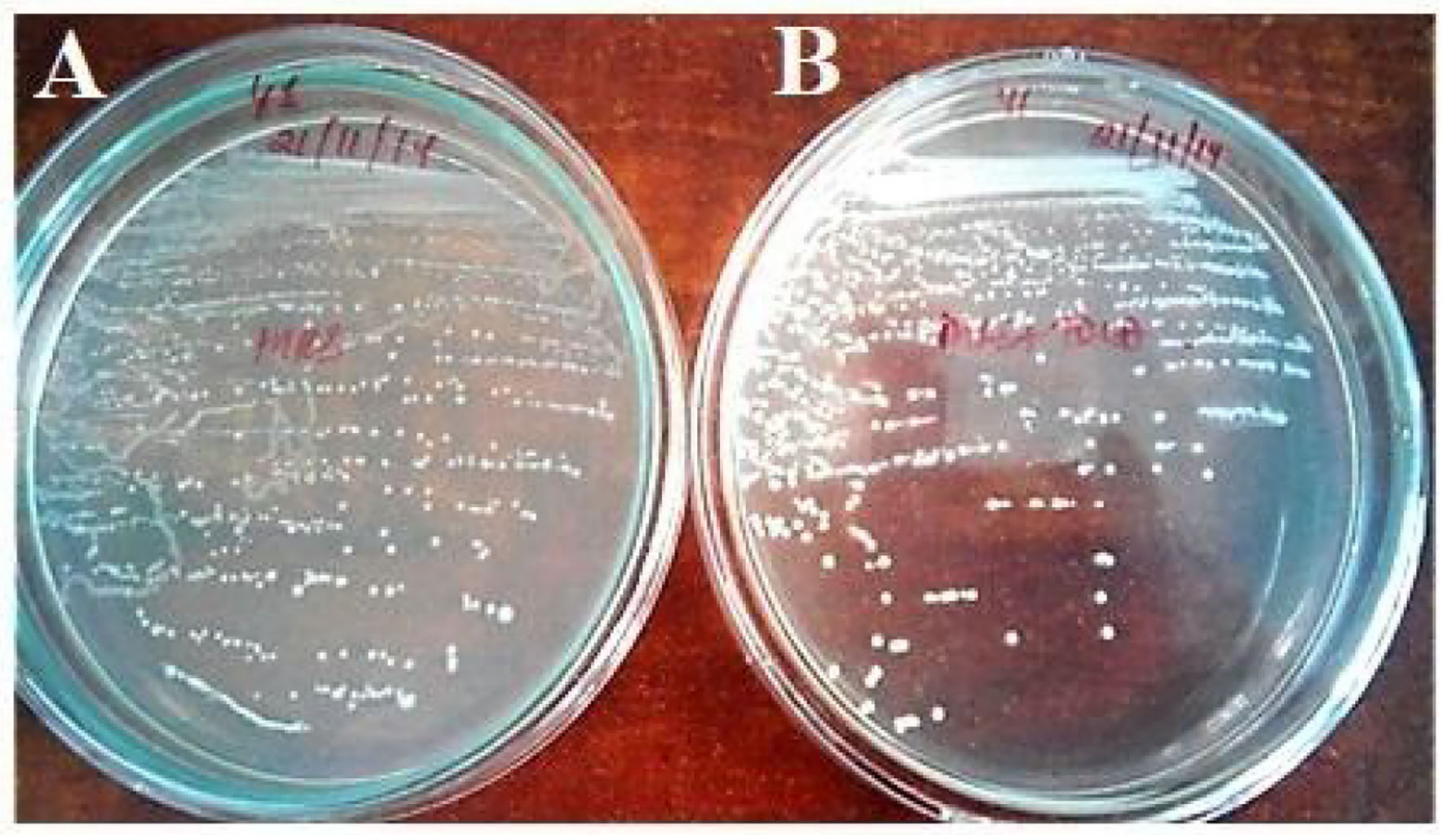
Bile salt hydrolase activity of *Enterococcus lactis* YY1, **(A)** On MRS plate and **(B)** MRS plate supplemented with TDCA.

The strain *Enterococcus lactis* YY1 was more acid-tolerant than *Enterococcus durans* HS03 showing 6.47–7.15 log cycles in comparison to 6.6 log cycle at pH 2.5 for 2 h. Then, on testing the isolate pH 2, it was observed that both strains could tolerate the acidic condition for up to 2 h. Tolerance to three bile salts: ox bile, cholic acid, and taurocholic acid at 0.5 and 1% concentrations were studied. The results were calculated as the percentage increase in turbidity, in the case of *Enterococcus lactis* YY1 94, 892, and 107% in 0.5% ox bile, cholic acid, and taurocholic acid and 94, 977, and 83% in 1% bile salts were observed, respectively. In *Enterococcus durans* HS03 percentage increase in turbidity at 0.5% bile concentration was 277, 1,014, and 62% and 169, 627, and 100% at 1%. Both the strains showed good cell surface hydrophobicity. *Enterococcus lactis* YY1 was BSH-positive and showed excellent BSH enzyme activity ^*^(Table 4) in sodium taurocholate and sodium glycocholate (Figure 1). *Enterococcus durans* HS03 was BSH-negative although it is a cholesterol-lowering strain that tolerates bile acid concentration.

**Table 4:** BSH activity of *Enterococcus lactis* YY1.

**Sodium glycocholate**			**Sodium taurocholate**		
**Total protein mg/ml**	**Total activity U/ml**	**Specific activity U/mg**	**Total protein mg/ml**	**Total activity** **U/ml**	**Specific activity U/mg**
1.21 ± 0.11	1.30 ± 0.10	1.07 ± 0.11	1.22 ± 0.11	0.93 ± 0.11	0.76 ± 0.23

### Safety Evaluation of Probiotics for Human Use

**Figure 2:**
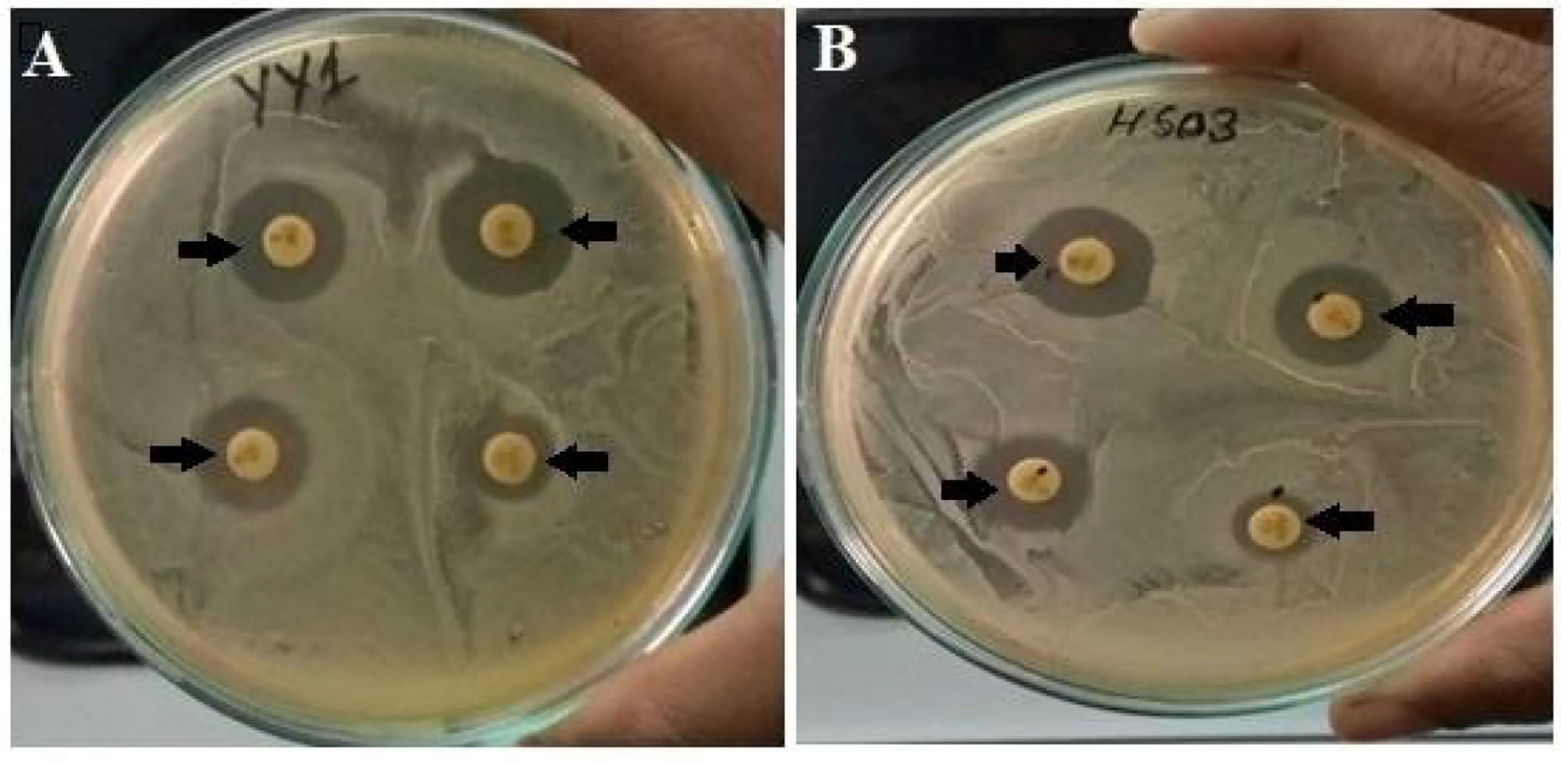
**(A)** Antibiotic sensitivity pattern of *Enterococcus lactis* YY1 and **(B)** *Enterococcus durans* HS03.

The strains were resistant to penicillin, however, the highest zone of inhibition was seen in both the strains against vancomycin and azithromycin antibiotics (Table 5; Figure 2).

**Table 5:** Antibiotic sensitivity test.

**Strain**	**Inhibition zone in mm**							
	**P**	**E**	**VA**	**TEI**	**CD**	**OF**	**AZM**	**TE**
*Enterococcus lactis* YY1	–	25-S	23 - S	17- I	17-I	16-I	17-I	32-S
*Enterococcus durans* HS03	–	25-S	23-S	16-I	16-I	16-I	10-I	30-S

*Results were expressed as sensitive, S (≥21 mm), intermediate, I (16–20 mm) and Resistant, R (≤ 15 mm), respectively, according to that described by Charteris et al. (1998) and Volkova et al. (2013). The strains were sensitive to penicillin. Penicillin (P) 10 units, erythromycin (E) 15 μg, vancomycin (VA) 30 μg, teicoplanin (TEI) 30 μg, clindamycin (CD) 2 μg, ofloxacin (OF) 5 μg, azithromycin (AZM) 15 μg and tetracycline (TE) 30 μg*.

**Figure 3:**
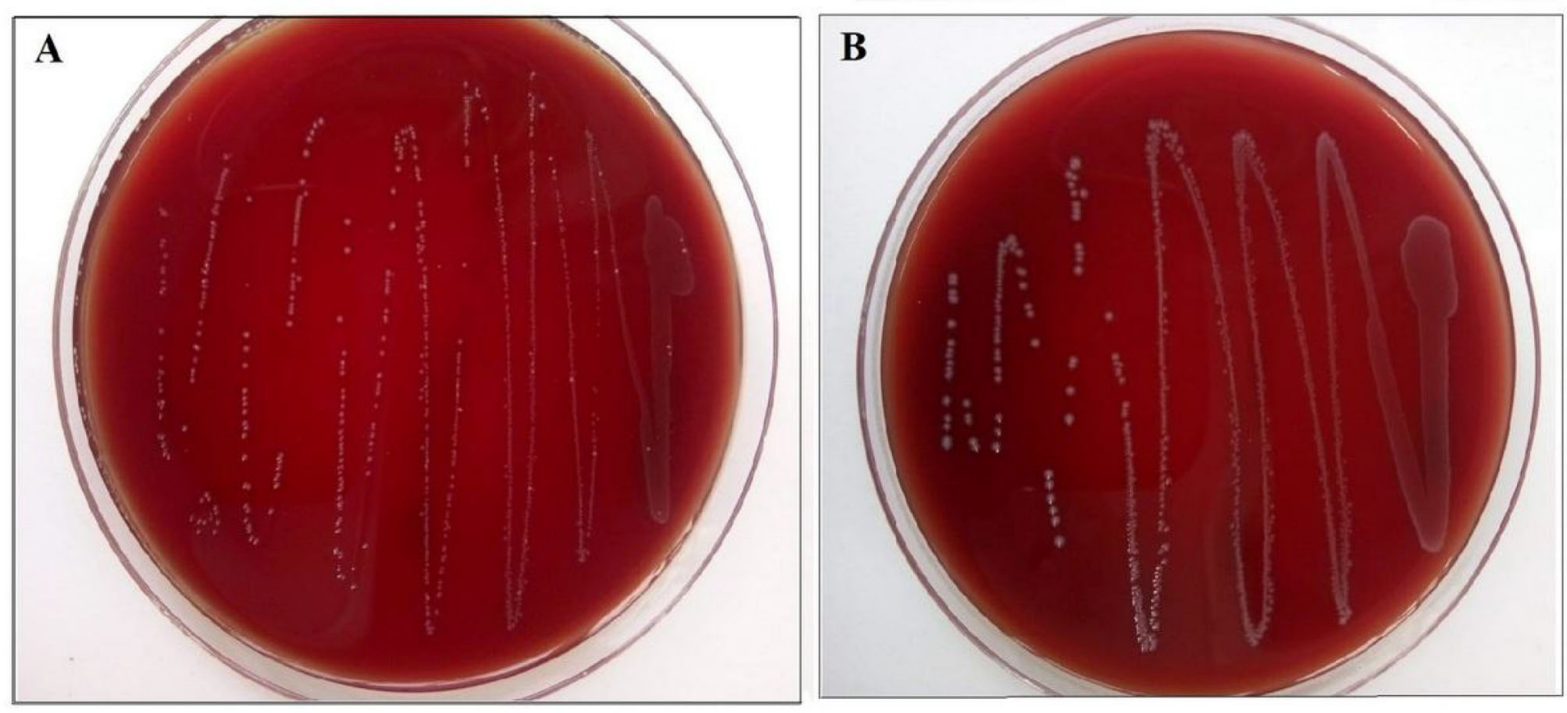
**(A)** *Enterococcus lactis* YY1 and **(B)** *Enterococcus durans* HS03 showing negative hemolytic activity on sheep blood agar.

The strains gave no reaction on sheep blood agar, hence both strains gave a negative test for hemolytic activity (Figure 3).

### Bodyweight and Feed Efficiency

**Figure 4:**
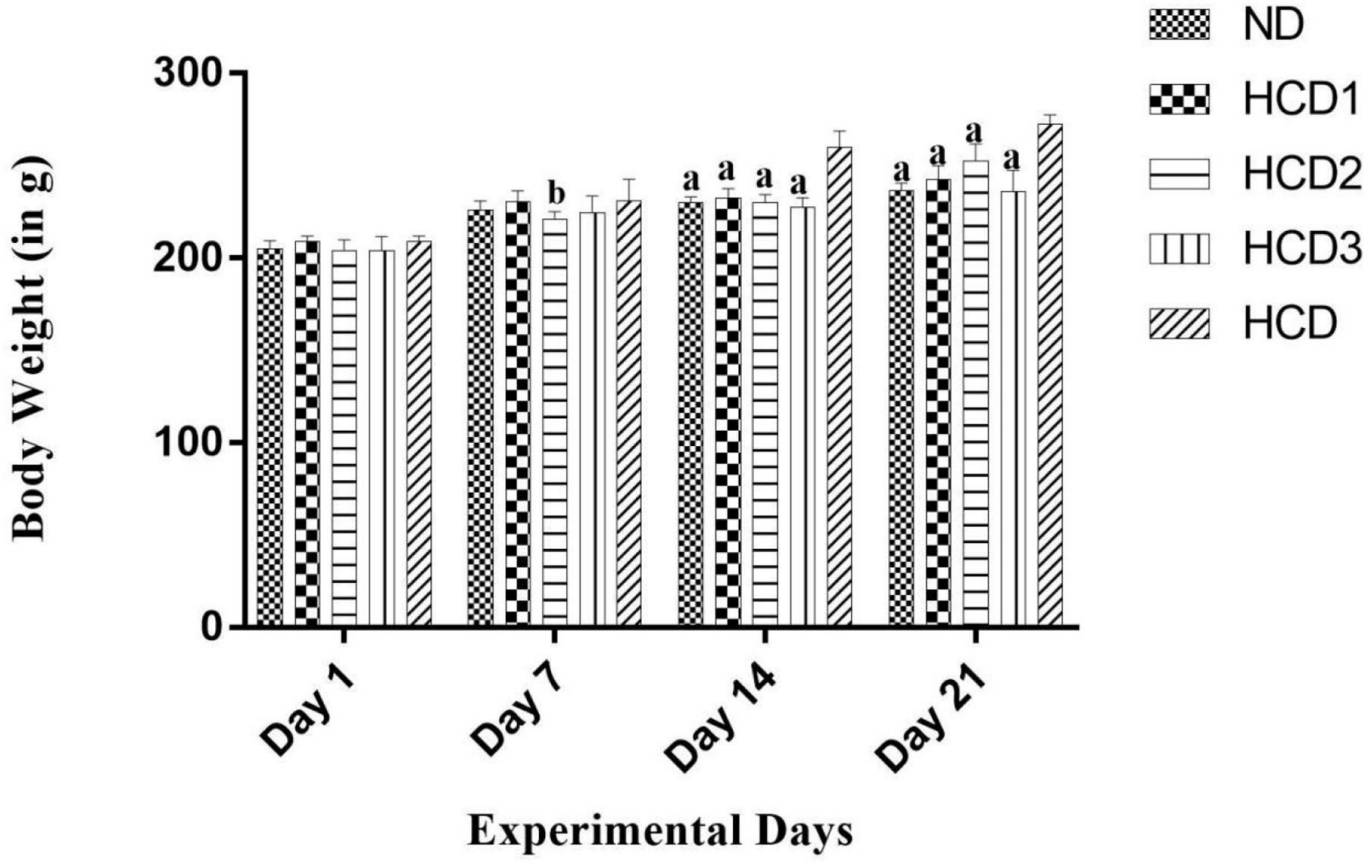
Bodyweight increment in all experimental groups after 21 days. Results are shown as mean ± SEM (*n* = 6). “a” denotes *p* ≤ 0.0001; “b” denotes *p* ≤ 0.001 vs. Hypercholesterolemic diet control (HCD) group.

All the experimental rats used in the study remained healthy, and their body weight gain and feed intake after 21 days were calculated and recorded for all the groups in Table 6. The statistical analysis showed a significant (*p* < 0.05) difference in final body weight and gain in weight among the experimental groups when compared to the hypercholesterolemic diet groups. After 3 weeks of treatment, the final bodyweight of the normal diet control group (ND) and the probiotic-treated groups (HCD1, HCD2, and HCD3) was 236.5 ± 1.708, 242.5 ± 3.096, 252.5 ± 3.819, and 236 ± 4.726 gm, respectively. The hypercholesterolemic diet control (HCD) group which was fed with a cholesterol-enriched diet, showed a significantly (*p* < 0.05) higher body weight (272.5 ± 2.141 gm) in comparison to other experimental groups (Figure 4). The highest body weight gain was also recorded in the HCD group (63.75 ± 5.42 gm), while the body weight gain of all the other experimental groups seems to be recorded significantly lower (*p* < 0.05) than the HCD group. Similarly, feed intake was higher in the HCD group (208.2 ± 2.643 gm) than in probiotic-treated groups (HCD1, HCD2, and HCD3) and the normal diet control group (ND) group. The feed intake of the normal diet control group (ND) and the probiotic-treated groups (HCD1, HCD2, and HCD3) was 180.8 ± 6.193, 186 ± 3.813, 202.3 ± 3.163, and 182 ± 5.366 gm, respectively, which was significantly lower (*p* < 0.05) than the HCD group.

**Table 6:** Bodyweight, weight gain, and feed intake of rats fed with experimental diets (mean values, *n* = 6).

**Group**	**Initial weight (g)**	**Final weight (g)**	**Weight gain (g)**	**Feed intake (g)**
ND	205 ± 1.826	236.5 ± 1.708^***^	31.5 ± 2.432^***^	180.8 ± 6.193^***^
HCD1	208.75 ± 1.25	242.5 ± 3.096^***^	33.75 ± 3.146^***^	186 ± 3.813^**^
HCD2	204 ± 2.38	252.5 ± 3.819^***^	48.5 ± 4.89^**^	202.3 ± 3.163^ns^
HCD3	204 ± 3.109	236 ± 4.726^***^	32 ± 7.113^***^	182 ± 5.366^***^
HCD	208.75 ± 1.25	272.5 ± 2.141	63.75 ± 5.42	208.2 ± 2.643

*Analysis was done by One-way ANOVA followed by Dunnett's post hoc test where*

*** *indicates p ≤ 0.001,*

** *indicates p ≤ 0.01, and*

ns *indicates non-significant value*.

### Analysis of Serum Lipid Profile

**Figure 5:**
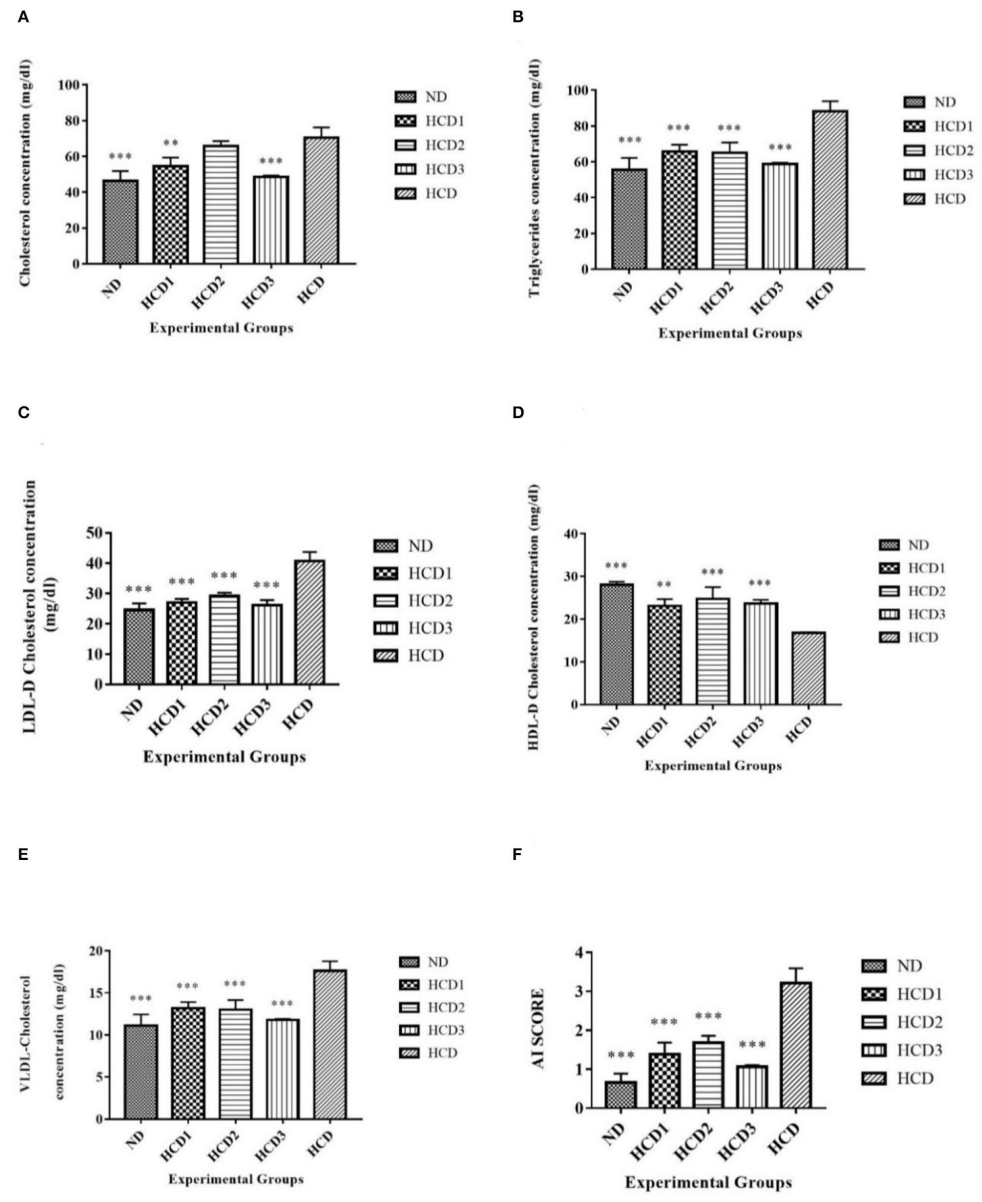
Serum lipid parameters of different experimental groups. Analysis was done by One-way ANOVA followed by Dunnett's *post-hoc* test where ^***^indicates *p* ≤ 0.001 and ^**^indicates *p* ≤ 0.01. All data represented as mean ± SD of 6 animals. **(A)** Cholesterol **(B)** Triglycerides. **(C)** LDL-D cholesterol. **(D)** HDL-D cholesterol. **(E)** VLDL-D cholesterol. **(F)** Atherogenic Index (AI).

The effect of dietary treatments on serum lipid profile (serum total cholesterol, TG, LDL-cholesterol, HDL-cholesterol, VLDL-cholesterol, and AI) has been recorded in Table 7 and Figure 5.

**Table 7:** Serum total cholesterol, triglyceride, LDL-, HDL-D-cholesterol, VLDL and AI score in experimental rats.

**Group**	**Total cholesterol (mg/dl)**	**Triglyceride (mg/dl)**	**LDL-cholesterol (mg/dl)**	**HDL-cholesterol (mg/dl)**	**VLDL (mg/dl)**	**Atherogenic index (AI) score**
ND	46.4 ± 5.45^***^	55.5 ± 6.68^***^	24.6 ± 2.09^***^	28 ± 0.736^***^	11.1 ± 1.34^***^	0.659 ± 0.224^***^
HCD1	54.6 ± 4.77^**^	65.8 ± 3.66^***^	27 ± 1.27^***^	23.1 ± 1.6^**^	13.2 ± 0.732^***^	1.38 ± 0.304^***^
HCD2	65.8 ± 2.86^ns^	65 ± 5.74^***^	29.1 ± 1.13^***^	24.7 ± 2.76^***^	13 ± 1.15^***^	1.67 ± 0.185^***^
HCD3	48.6 ± 0.827^***^	58.9 ± 0.659^***^	26.1 ± 1.76^***^	23.7 ± 0.871^***^	11.8 ± 0.132^***^	1.05 ± 0.0514^***^
HCD	70.5 ± 5.67	88.2 ± 5.62	40.7 ± 2.98	16.7 ± 0.20	17.6 ± 1.12	3.21 ± 0.372

*Analysis was done by One-way ANOVA followed by Dunnett's post hoc test where*

*** *indicates p ≤ 0.001,*

** *indicates p ≤ 0.01, and*

ns *indicates non-significant value*.

**Total serum cholesterol** values for each dietary treatment group differed significantly (*p* < 0.05) and were recorded as 46.4 ± 5.45, 54.6 ± 4.77, 65.8 ± 2.86, 48.6 ± 0.827, and 70.5 ± 5.67 mg/dl for the ND, HCD1, HCD2, HCD3, and HCD dietary groups, respectively (Figure 5A). The dietary treated groups HCD1, HCD2, and HCD3 showed decreases in serum cholesterol levels up to 22.55, 6.67, and 31.06%, respectively, when compared with the HCD group after 21 days of dietary treatment.

The **serum TG** and **VLDL-cholesterol** concentrations also differed significantly (*p* < 0.05) among all the groups throughout the experiment. After dietary treatment, TG levels for all the five groups were recorded as 55.5 ± 6.68, 65.8 ± 3.66, 65 ± 5.74, 58.9 ± 0.659, and 88.2 ± 5.62 mg/dl for the ND, HCD1, HCD2, HCD3, and HCD groups, respectively (Figures 5B,E). The reduction in TG and VLDL concentrations was 25.39, 26.3, and 33.21% in the HCD1, HCD2, and HCD3 groups, respectively, when compared with the HCD group.

**LDL-cholesterol** values for each dietary treatment group also differed significantly (*p* < 0.05) and were recorded as 24.6 ± 2.09, 27 ± 1.27, 29.1 ± 1.13,26.1 ± 1.76, and 40.7 ± 2.98 mg/dl for the ND, HCD1, HCD2, HCD3, and HCD groups, respectively (Figure 5C). The reduction in LDL-cholesterol was 33.66, 28.50, and 35.87% in the HCD1, HCD2, and HCD3 groups, respectively, when compared with the HCD group.

The **HDL-cholesterol** values for all the five groups were recorded as 28 ± 0.712, 23.1 ± 1.6, 24.7 ± 2.76, 23.7 ± 0.871, and 16.7 ± 0.20 mg/dl for the ND, HCD1, HCD2, HCD3, and HCD groups, respectively (Figure 5D). There is a significant difference (*p* < 0.05) among the HDL-cholesterol values of different experimental groups. The resulting increase of HDL-cholesterol in the probiotic treatment groups, i.e., HCD1, HCD2, and HCD3 was 38.32, 47.9, and 41.92%, respectively, when compared with the HCD group.

The **Atherogenic Index (AI) score** of the dietary treatment groups of the rats also differed significantly (*p* < 0.05). The AI score of the ND group was 0.659 ± 0.224. However, the AI score of the probiotic-treated groups was found to decrease after 21 days of treatment as compared with the HCD group (3.21 ± 0.372). The maximum reduction in AI score was recorded in the HCD3 group (1.05 ± 0.0514) than in the HCD1 (1.38 ± 0.304) and HCD2 groups (1.67 ± 0.185) (Figure 5F).

### Changes in Liver Total Cholesterol and Triglyceride

**Figure 6:**
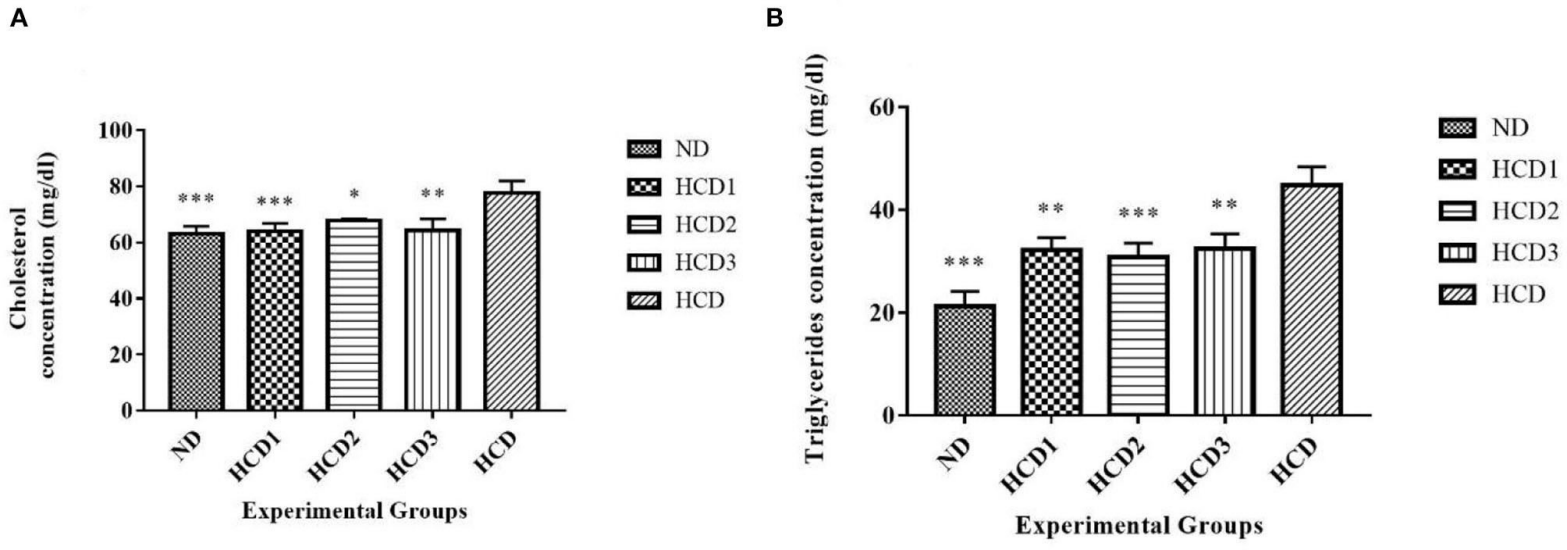
Liver lipid parameters of different experimental rats. Analysis was done by One-way ANOVA followed by Dunnett's *post-hoc* test where ***indicates *p* ≤ 0.001, **indicates *p* ≤ 0.01, and *indicates *p* ≤ 0.05. All data represented as mean ± SD of 6 animals. **(A)** Cholesterol (liver). **(B)** Triglycerides (liver).

The **total cholesterol level** significantly (*p* < 0.05) differed among the supernatants of liver homogenate among different experimental groups. The **total cholesterol level** of different experimental groups was recorded as 63.1 ± 2.76, 64 ± 2.83, 67.8 ± 0.527, 64.2 ± 4.13, and 77.6 ± 4.35 mg/dl for the ND, HCD1, HCD2, HCD3, and HCD dietary groups, respectively (Figure 6A). The dietary treated groups HCD1, HCD2, and HCD3 showed decreases in serum cholesterol levels up to 17.52, 12.63, and 17.16%, respectively, when compared with the HCD group after 21 days of dietary treatment.

The **triglyceride (TG)** concentrations from the supernatants of liver homogenate also differed significantly (*p* < 0.05) among all the groups throughout the experiment. After dietary treatment, **TG levels** for all the five groups were recorded as 21.2 ± 2.97, 32.1 ± 2.47, 30.8 ± 2.71, 32.4 ± 2.92, and 44.8 ± 3.62 mg/dl for the ND, HCD1, HCD2, HCD3, and HCD groups, respectively (Figure 6B). The reduction in TG concentrations was 28.35, 31.25, and 27.68% in the HCD1, HCD2, and HCD3 groups, respectively, when compared with the HCD group.

### Effects on Liver and Kidney Histopathology

**Figure 7:**
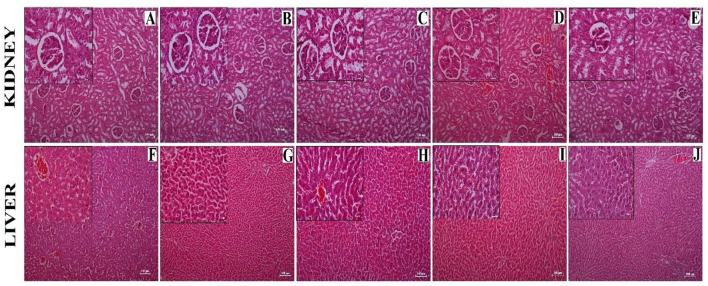
Histological sections showing kidney **(A–E)** and liver **(F–J)** obtained from different experimental groups. **(A,F)** = Normal diet control (ND) group, **(B,G)** = HCD1 group, **(C,H)** = HCD2 group, **(D,I)** = HCD3 group, and **(E,J)** = Hypercholesterolemic diet control (HCD) group. The main images represent sections observed under 10X magnification; the inset represents 40X magnified images. Scale bars of 100 and 25 μm are allotted for 10X and 40X magnifications, respectively.

From the histopathology, no observable structural changes were found in kidney sections between the different experimental groups. The sections of kidneys (Figures 7A–E) were found to have well-organized Bowman's capsule cells (Black arrow). There was no significant structural change observed between the experimental groups. A moderate degree of fatty vacuolization was observed in the HCD treated group (Figure 7J). However, the liver sections (Figures 7F–I) of all the experimental groups including the normal control group showed well-organized cellular (lobule) structure and no abnormalities were seen in any groups than the HCD-treated group (Figure 7J).

### Effect on Liver and Body Lipid Index

**Figure 8:**
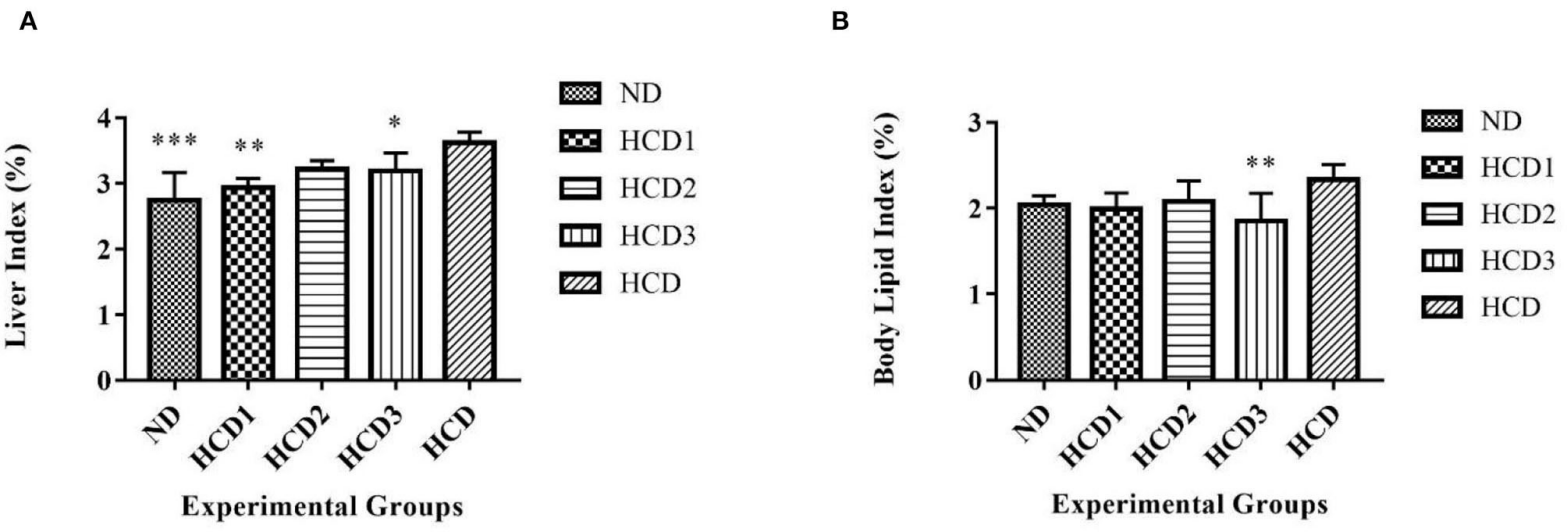
Liver and organ lipid indexes in different experimental rat groups. Analysis was done by One-way ANOVA followed by Dunnett's *post-hoc* test where ***indicates *p* ≤ 0.001, **indicates *p* ≤ 0.01, and *indicates *p* ≤ 0.05. All data represented as mean ± SD of 6 animals. **(A)** Liver index. **(B)** Body lipid index.

The liver index was found to be significantly low in the ND, HCD1, HCD2, and HCD3 groups than the HCD group (2.74 ± 0.429%, 2.93 ± 0.141%, 3.22 ± 0.13%, 3.19 ± 0.271% vs. 3.62 ± 0.158%, *p* < 0.05) (Figure 8A). The body lipid index also showed significant difference among the experimental groups (*p* < 0.05). The highest percentage of body lipid index was recorded in the HCD group (2.34 ± 0.177%), while the lowest percentage was recorded in the HCD3 group (1.85 ± 0.326%, *p* = 0.007) (Figure 8B).

### Effects of Probiotic Strains on Oxidative Stress in the Liver of Rats

**Figure 9:**
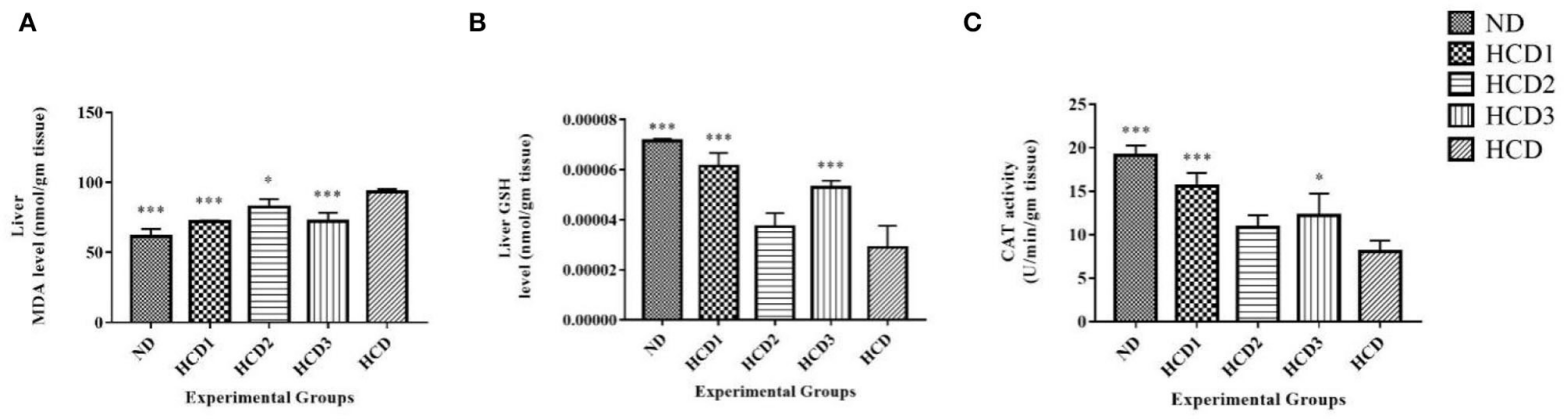
Effect of probiotic strains on MDA, GSH, and CAT levels in the liver of high-fat diet fed rats. **(A)** Liver MDA level. **(B)** Liver GSH level. **(C)** Liver CAT activity. Analysis was done by One-way ANOVA followed by Dunnett's *post-hoc* test where ***indicates *p* ≤ 0.001 and *indicates *p* ≤ 0.05. All data represented as mean ± SD of 6 animals.

Long-term intake of hypercholesterolemic diet results in obesity and increased oxidative stress in the liver. Oxidative stress is assessed by measuring the malondialdehyde (MDA), reduced glutathione (GSH), and catalase (CAT) activities in the liver. The effects of the probiotic strains on the level of MDA as well as the activities of the other antioxidant enzymes in the liver are presented in Figure 9.

The MDA levels in the hypercholesterolemic diet (HCD) group were significantly (*p* < 0.05) higher than that of the normal control group (by a 3.42-fold increase) and other probiotic strain-fed groups, indicating the over-peroxidation of liver injury (Figure 9A). The lowest MDA level was recorded in the HCD1 and HCD3 groups which were fed with probiotic strain 1 and a combination of both probiotic strains 1 and 2, respectively, as compared with the negative control group (HCD).

At the same time, the liver GSH level showed a significant (*p* < 0.05) decrease in the hypercholesterolemic diet (HCD) group when compared to those of the normal control (ND) group as well as the probiotic strain-fed groups (Figure 9B). In the liver, supplementation of probiotic strains significantly (*p* < 0.05) increased the concentration of reduced-GSH. However, the reduced-GSH level recorded in the HCD1 and HCD3 groups (which were fed with probiotic strain 1 and a combination of both probiotic strains 1 and 2, respectively), showed the best results when compared with the hypercholesterolemic diet (HCD) group.

In this study, the catalase activity was significantly decreased in the hypercholesterolemic diet (HCD) group (Figure 9C) compared to the normal control rats. Probiotic supplement significantly (*p* < 0.05) restored the catalase activity in high-fat diet-fed rats to the levels of normal control rats (ND). The catalase activity recorded in the HCD1 group (fed with probiotic strain 1) showed the best results when compared with the hypercholesterolemic diet (HCD) group.

All these results suggest that treatment with probiotic strains could improve the hepatic antioxidant status.

### Fecal Sample Analysis

SD rats fed with probiotic supplements showed comparatively higher total bacterial counts in their feces than that of the control groups. Their fecal total bacterial count ranged from 8.273 to 8.900 CFU log_10_/g. Also, a higher number of lactobacilli counts, ranging from 8.263 to 8.830 CFU log_10_/g was recorded in the feces of the SD rats fed with probiotic supplements in comparison to the control groups. Further, the experimental groups HCD1 and HCD3 showed maximum lactobacilli count in their feces among all the other groups. In contrast to the total bacterial counts and the lactobacilli counts in the feces of the SD rats fed with probiotics, their fecal coliform and *E*. *coli* counts decreased relative to that of the control groups. Alternatively, the maximum fecal coliform and *E*. *coli* count were observed in the HCD group. The HCD fecal coliform and *E*. *coli* count ranged from 7.633 to 8.100 and 7.653 to 8.350, respectively. The overall mean value and the ±SEM of the bacterial counts in the experimental SD rat feces on days 0, 7, 14, and 21 procured from distinct experimental groups have been tabulated in Table 8.

**Table 8:** Effect of feeding probiotics on fecal bacterial population (*n* = 3).

**Log CFU/g of feces**	**Day**	**ND**		**HCD1**		**HCD2**		**HCD3**		**HCD**	
		**Mean**	**SEM**	**Mean**	**SEM**	**Mean**	**SEM**	**Mean**	**SEM**	**Mean**	**SEM**
Total aerobes	0	8.273	0.018	8.273	0.018	8.273	0.018	8.273	0.018	8.273	0.018
	7	8.233^ns^	0.038	8.657^a^	0.038	8.720^a^	0.035	8.827^a^	0.043	8.207	0.032
	14	8.227^ns^	0.037	8.673^a^	0.058	8.723^a^	0.034	8.823^a^	0.039	8.250	0.025
	21	8.187^ns^	0.026	8.647^a^	0.026	8.710^a^	0.023	8.900^a^	0.055	8.150	0.017
*Lactobacillus*	0	8.270	0.072	8.270	0.072	8.270	0.072	8.270	0.072	8.270	0.072
	7	8.110^a^	0.023	8.603^a^	0.033	8.263^a^	0.071	8.633^a^	0.041	7.627	0.041
	14	8.247^ns^	0.032	8.647^a^	0.044	8.460^b^	0.070	8.750^a^	0.017	8.237	0.035
	21	8.260^c^	0.023	8.830^a^	0.012	8.470^a^	0.029	8.757^a^	0.015	8.060	0.029
Coliforms	0	7.633	0.035	7.633	0.035	7.633	0.035	7.633	0.035	7.633	0.035
	7	7.660 ^ns^	0.064	7.453^b^	0.049	7.563^ns^	0.045	7.420^a^	0.031	7.633	0.041
	14	7.560^a^	0.035	6.830^a^	0.040	8.030^ns^	0.029	7.437^a^	0.023	8.100	0.029
	21	7.600^a^	0.029	6.360^a^	0.023	7.570^a^	0.017	6.450^a^	0.017	8.450	0.017
*Escherichia coli*	0	7.653	0.047	7.653	0.047	7.653	0.047	7.653	0.047	7.653	0.047
	7	7.660^ns^	0.069	7.130^a^	0.029	7.583^c^	0.009	7.400^a^	0.042	7.733	0.073
	14	7.467^a^	0.009	6.437^a^	0.034	7.597^a^	0.026	7.423^a^	0.035	8.010	0.040
	21	7.670^a^	0.017	7.090^a^	0.035	7.520^a^	0.023	7.427^a^	0.044	8.350	0.029

*ND, normal diet; HCD1, hypercholesterolemic diet containing Enterococcus; HCD2, hypercholesterolemic diet containing Enterococcus; HCD3, hypercholesterolemic diet containing Enterococcus durans; HS03, Enterococcus lactis YY1*.

a *p < 0.0001 and < 0.001*.

b *p < 0.01*.

c *p < 0.05*.

ns *indicates non-significant value*.

## Discussion

*Enterococcus* sp. are highly diverse and are present as common microbiota in the environment, the intestine of humans, mammals, and fermented foods (Giraffa, 2002; Li et al., 2018; Zommiti et al., 2018). A similar type of lactic acid bacteria such as *Enterococcus, Lactococcus, Lactobacillus, Leuconostoc* were also reported in ethnic starter cultures of Sikkim by Sha et al. (2017, 2018, 2019). There has been research on the probiotic properties of lactic acid bacteria like *Lactobacillus* spp. and *Bifidobacterium* but only limited studies have been undertaken on the probiotic attributes of *Enterococcus* spp. Therefore, in this study, the *Enterococcus* strains, namely *Enterococcus durans* HS03 (accession no. KX274030) and *Enterococcus lactis* YY1 (accession no. KU 601443) (Ghatani and Tamang, 2017) were studied for their probiotic properties, especially cholesterol-lowering, as one of the important health benefits by *in vivo* studies, which makes it one of the first kind of studies conducted so far. The strains were previously isolated from fermented yak milk, a product of the Sikkim Himalayas known as *chhurpi* and healthy human gut, and then tested for cholesterol reduction and were identified by 16s rDNA sequencing (Ghatani and Tamang, 2017). A probiotic bacterium should have at least one health benefit. In human blood, higher than 1 mmol of cholesterol (i.e., 18 mg/dl) than the normal level is reported to increase the risk of coronary heart disease and coronary death. There is growing public attention to healthy food due to increasing knowledge in society as statins that lower high cholesterol are expensive and are reported to cause severe side effects (Golomb and Evans, 2008).

For a strain to be considered as a potential probiotic candidate, it should be able to tolerate or resist the pH conditions, bile salt concentrations, and cell surface hydrophobicity. The beneficial bacteria through the oral route pass through the highly acidic gastric juice in the stomach (approximately pH 3) and a weakly basic juice in the intestine (pH of 7.8–8.4) containing 0.3–2% (w/v) bile salts in the upper part of the intestinal tract where they can stay for 1–2 h (Chen et al., 2010). The strains were first tested at pH 2.5 and pH 2 which is considered to be a strong differential pH range (Turchi et al., 2013). Both the strains were observed to tolerate pH up to 2 h in both pH ranges as in accordance to the tolerance reported in the case of *Lactobacillus strains* (Liong and Shah, 2005). However, similar to our study, a decrease in pH with time has also been reported by Raghavendra et al. (2010).

The ability to tolerate bile concentrations studied by different researchers varies in concentration, however, we had used oxgall, taurocholic acid, and cholic acid at 0.5 and 1% for 0, 4, and 8 h, in accordance to 0.5% (Mathara et al., 2008), 0.1–0.3% (Dunne et al., 2001) and the prevailing time is suggested to be 4 h (Mishra and Prasad, 2005). The two strains showed variability concerning acid and bile tolerance which may be due to species and strain specificity.

Cell surface hydrophobicity is one of the important criteria as probiotics need to be adhered to the intestinal mucosa to avoid being removed from the peristaltic movement of the colon. *Enterococcus durans* HS03 and *Enterococcus lactis* YY1 gave 65 and 70% cell surface hydrophobicity percentage, respectively, which was as per the report where hydrophobic index > 40% was considered as hydrophobic (Boris et al., 1998) and Nostro et al. (2004) recommended hydrophobicity index > 70% for adherence to the cell surfaces; 38.1–67.8% hydrophobicity was observed by *L. acidophilus* (Vinderola and Reinheimer, 2003) which was in accordance with our study shown by *Enterococcus durans* HS03.

Several mechanisms for cholesterol-lowering have been reported like cholesterol assimilation, cholesterol co-precipitation with deconjugated bile, binding of cholesterol to the bacterial cell wall, and enzymatic deconjugation of bile acids due to the presence of BSH enzyme. The mechanism for cholesterol-lowering in our study was the presence of the BSH enzyme which was similar to Liong and Shah, 2005 and Lye et al. (2010). Numerous lactic acid bacterial species like *Lactobacillus* spp., *Bifidobacterium* spp., *Streptococcus* spp., and *Pediococcus* spp. with bile salt hydrolase (BSH) activity have been studied and thereby recommended for their cholesterol-lowering effects (Lim et al., 2004; Zhang et al., 2017). One strain *Enterococcus lactis* YY1 was BSH-positive when checked in direct assay containing TDCA similar to those reported by Archer and Halami (2015) on screening the isolates from dairy and human fecal matter. The BSH enzyme-specific enzyme activity U/mg was more in the case of sodium glycocholate than sodium taurocholate as in earlier reports of BSH enzymes of *Lactobacillus* showing more affinity toward glycine-conjugated bile salts (Liong and Shah, 2005; Pavlović et al., 2012).

Antibiotic susceptibility of LAB is one of the important criteria for the safety of probiotics. *Enterococcus* species have been reported to become resistant to various antibiotics in some studies (Vidhyasagar and Jeevaratnam, 2013). Some probiotic enterococci transfer the antibiotic resistance to the nearby microbes in the gut and thereby disrupt the original intestinal flora (Taheur et al., 2016). Both the strains were susceptible to all antibiotics except penicillin which may be plasmid-borne or associated with the chromosomal DNA. According to Danielsen and Wind (2003), the resistance is due to the presence of D-Ala-D-lactate in their peptidoglycan instead of D-Ala-D-Ala dipeptide which is the target of the antibiotic.

The strains showed no hemolytic activity; thus, they were γ hemolytic, and the absence of hemolytic activity is considered a safety prerequisite for the selection of a probiotic strain. None of the strains showed α and β hemolytic activity when grown on sheep blood agar. Our study was similar to the observations made for the strains of *L. paracasei* subsp. *paraccasei, Lactobacillus* spp., and *L. casei* isolated from the dairy products (Maragkoudakis et al., 2006) and foods (Kumar and Murugalatha, 2012).

To determine the relationship between the administration of different strains of *Enterococcus* and the status of serum lipid profiling, *in vivo* study using the high-fat diet-induced obesity model in SD rats was done. Obesity is typically associated with body weight and dyslipidemia, which encompasses elevated levels of TC, TG, and LDL-C as well as lowered HDL-C levels. All of these are risk factors for cardiovascular disease (Skrypnik et al., 2018; Wang et al., 2021). The long-term exposure of high-fat diet seems to have a significant relation to the onset of obesity from various rat model studies (Yin et al., 2010; Lasker et al., 2019). From our results, the onset of obesity was also supported by the body weight and feed efficiency data. The hypercholesterolemic diet control (HCD) group which was fed with a cholesterol-enriched diet recorded the highest body weight gain increase due to the increased feed consumption. However, upon treatment with the probiotic strain, the HCD3 group, which was fed with both the *Enterococcus durans* HS03 and *Enterococcus lactis* YY1 strains significantly lowered its body weight as well as its feed intake when compared to the hypercholesterolemic diet control (HCD) group. The body lipid index data also follows a similar trend. The HCD3 group records the lowest body lipid index, when compared to the HCD group, indicating the effect of both *Enterococcus durans* HS03 and *Enterococcus lactis* YY1 strains in the amelioration of obesity in these experimentally induced obese rats. It was evident that different strains of *Enterococcus* might act as a new therapeutic probiotic candidate in controlling body weight gain.

Under normal conditions, the liver, which is an important site of lipid metabolism in the body, maintains a balance of lipid synthesis and decomposition, whereas a high-fat diet disrupts this balance, causing excessive lipid accumulation (i.e., hepatic steatosis) and oxidative stress in the liver, indicating the presence of liver injury (Skrypnik et al., 2018; Wang et al., 2021). Accumulation of lipids in the liver is the main cause of non-alcoholic fatty liver disease (NAFLD), which is a common complication of obesity (Skrypnik et al., 2018). Furthermore, multiple studies have shown that obesity is directly connected with body-liver weight, liver TC and liver TG, and serum lipid profiles in high-fat diet-induced obese rats (Skrypnik et al., 2018; Wang et al., 2021; Yang et al., 2021). In our experiments, it was evident that the high-fat diet-fed rats (HCD rats) showed an increased TC and TG levels from both the serum and liver tissue homogenate, which further triggered the lipid accumulation and lipotoxicity in the liver. The levels of TC, TG, LDL-C, and HDL-C in the serum of the different experimental groups of SD rats were evaluated after 3 weeks of animal feeding to assess their blood lipid metabolism. The serum levels of TC, TG, and LDL-C were dramatically raised in the HCD-treated group compared to the control group, while serum levels of HDLC were significantly lowered, as expected (Table 6). However, the *Enterococcus* treatment on the experimental rat groups, on the other hand, restored these abnormalities in SD rats fed on a high-fat diet, suggesting a reduction in metabolic dysfunction. This reduction of serum lipid profile may be due to the assimilation and reduced reabsorption of cholesterol by bacterial strain, deconjugation of bile acids by *Enterococcus* bile salt acid hydrolase, and increase cholesterol uptake by low-density lipoprotein receptor pathway in the liver tissues (Gill and Guarner, 2004). Moreover, as evidenced by liver histology, the degree of fatty accumulation in the liver was significantly reduced in all intervention experimental groups.

Long-term high-fat diets are an inappropriate dietary structure, and oxidative stress may result from increased reactive oxygen species (ROS) formation in the mitochondria of liver tissues due to fatty acid oxidation. Although oxidation activities are critical to life for energy and metabolism, excessive ROS can cause hepatic oxidative damage and may also trigger fibrosis in liver tissue, posing a serious threat to physical health, particularly in obese patients (Lasker et al., 2019). However, recent reports suggest that treatment with various probiotic strains, such as *Bifidobacterium animalis, Lactobacillus rhamnosus*, and *Bacillus* LBP32 significantly induce potent antioxidant capacity and reduce the oxidative damages *in vivo* models (Wu et al., 2019). MDA is a major byproduct of lipid peroxidation and also serves as an efficient reliable biomarker for oxidative stress-induced liver injury (Lin et al., 2018). The liver is a major organ involved in lipid metabolism. It was reported that various *Lactobacillus* strains can reduce the increase of MDA levels in the liver induced by D-gal (Lin et al., 2018). In this study, the levels of lipid peroxidation in the liver of hypercholesterolemic diet control (HCD) group were significantly higher than the normal (ND) group. However, upon the treatment with *Enterococcus durans* HS03 and *Enterococcus lactis* YY1 strains, both the experimental groups (HCD1 and HCD2) significantly reduced the MDA levels in the liver of the experimental rats.

The animal body has been demonstrated to have an effective mechanism for preventing free radical-induced tissue cell damage, which is accomplished *via* a set of endogenous antioxidant enzymes and proteins such as GSH and CAT. GSH and CAT formed a defense against ROS that was mutually helpful. Studies have demonstrated that the antioxidant mechanisms of probiotics include chelating metal ion, possessing their own antioxidant enzymatic systems, and producing metabolites with antioxidative activity, such as GSH, butyrate, and folate (Wang et al., 2017). A recent report suggests that the yogurt supplementation increased the GSH and CAT levels in experimental rat models (Lasker et al., 2019). A similar pattern was also observed in our study. Supplementation of *Enterococcus durans* HS03 in the HCD1 group and both the strains of *Enterococcus durans* HS03 and *Enterococcus lactis* YY1 in HCD3 significantly increased the activities of the GSH, and CAT levels. This indicated that *Enterococcus* strains could keep scavenging free radicals and could improve the antioxidant defense system of hyperlipidemic rats.

Also, various pieces of literature have recorded the effects of feeding probiotic supplements on the fecal bacterial counts of animal models. However, the cholesterol-lowering effects of BSH-active *Enterococcus* spp. have scarcely been studied (Zhang et al., 2017). Further, quite a few studies have recorded the effects of feeding probiotic enterococci on the fecal bacterial count of animal models. As per our study, the microbial analysis of the SD rat feces revealed a significantly higher count of lactobacilli in the probiotic-fed SD rat in comparison to the control group rats. Lactobacillus has been considered the normal inhabitants of the intestinal microbiota in both humans as well as in animals. Also, they have been linked to various health benefits. In this study, SD rats supplemented with probiotic treatment showed higher total fecal lactobacilli counts as compared to that of the control SD rat groups, which is suggestive of the fact that feeding of the *Enterococcus* supplement has favored the intestinal stability of the lactobacilli. However, the lower coliform and *E. coli* count in the SD rats fed with probiotic supplements are on par with the findings previously recorded due to the probiotic feeding of experimental rats (Kumar et al., 2011). This finding is also on par with the study conducted by Zhu et al. (2019) and is suggestive of the fact that *E. durans* and *E*. *lactis* had a remarkable effect on the stability of the gut microflora, thereby indirectly assisting in cholesterol absorption and excretion (Zhu et al., 2019). The significant higher lactobacilli count in the probiotic-fed SD rats may also be attributed to the fact that lactic acid bacteria used in our study survived the low pH and high bile salt concentrations in the gastrointestinal (GI) tract, colonized the GI tract, and enhanced the intestinal probiotics (Kumar et al., 2011; Zhu et al., 2019). However, like in our findings, many previous reports have also revealed lower counts of *E*. *coli* and coliform in the feces of probiotic-fed rats (Kumar et al., 2011).

## Conclusion

Although the effects of probiotic bacteria have been investigated previously by different researchers, the literature specifically on the effect of BSH-positive and BSH-negative bacterial strains on fecal bacteria counts in animal models is quite less. Results of this study showed that both the *Enterococcus durans* HS03 and *Enterococcus lactis* YY1 strains possess probiotic properties *in vitro*. The supplementation of both the *Enterococcus durans* HS03 and *Enterococcus lactis* YY1 can improve the high-fat diet-related complications in experimental rat model based on a 21-day experimental schedule. Further, the serum lipid profiles, liver cholesterol, and triglycerides from all the experimental rat groups show the hypolipidemic properties of both the strains. The potential mechanism of this effect might be related to decreasing oxidative stress *via* several antioxidant indices. In this study, fecal microbial analysis showed significantly higher fecal lactobacilli counts in probiotic-treated groups compared to the control groups. This could be because of the ability of the strains to survive at low pH and high bile concentration as described previously *in vitro* experiments. Hence, in this study, *Enterococcus durans* HS03 and *Enterococcus lactis* YY1 which were investigated to explore probiotic properties reveal that these two LAB strains have the potential to be developed as probiotic foods for better health attributes. In this study, microencapsulation had not been conducted although microencapsulated probiotic strain reducing plasma cholesterol levels in rats had been reported by others. The result of this study reveals that consumption of *Enterococcus durans* HS03 and *Enterococcus lactis* YY1 as a probiotic dietary adjunct might be useful in reducing serum cholesterol levels in humans. However, placebo-controlled clinical trials need to be conducted to validate the efficacy and safety of the strain and its use in the management of high cholesterol.

## Data Availability Statement

The original contributions presented in the study are included in the article/supplementary material, further inquiries can be directed to the corresponding authors.

## Ethics Statement

The animal study was reviewed and approved by IAEC/NBU/2019/17 from CPCSEA (Committee for the Purpose of Control and Supervision of Experiments on Animals) of University of North Bengal, India.

## Author Contributions

KG conducted major experiments, analysis of data, and drafting of manuscript. ST has assisted in microbial analysis and drafting of the manuscript. DM and SS assisted with animal experiments, data analysis, and drafting of the manuscript. SPS was involved in developing the hypothesis, animal procurement, manuscript drafting, and manuscript finalizing. SB was involved in developing hypotheses, designing animal experiments, and approved of the final manuscript. All authors contributed to the article and approved the submitted version.

## Funding

The work was funded by the North Bengal University Research Grant 2019 and DST- SERB project (EEQ/2019/000738). The authors gratefully acknowledge the financial support of the University of North Bengal and DST- SERB project sanctioned to KG.

## Conflict of Interest

The authors declare that the research was conducted in the absence of any commercial or financial relationships that could be construed as a potential conflict of interest.

## Publisher's Note

All claims expressed in this article are solely those of the authors and do not necessarily represent those of their affiliated organizations, or those of the publisher, the editors and the reviewers. Any product that may be evaluated in this article, or claim that may be made by its manufacturer, is not guaranteed or endorsed by the publisher.
